# A Biophysical Basis for Mucus Solids Concentration as a Candidate Biomarker for Airways Disease

**DOI:** 10.1371/journal.pone.0087681

**Published:** 2014-02-18

**Authors:** David B. Hill, Paula A. Vasquez, John Mellnik, Scott A. McKinley, Aaron Vose, Frank Mu, Ashley G. Henderson, Scott H. Donaldson, Neil E. Alexis, Richard C. Boucher, M. Gregory Forest

**Affiliations:** 1 Cystic Fibrosis Pulmonary Research and Treatment Center, The University of North Carolina at Chapel Hill, Chapel Hill, North Carolina, United States of America; 2 Department of Physics and Astronomy, The University of North Carolina at Chapel Hill, Chapel Hill, North Carolina, United States of America; 3 Departments of Mathematics and Biomedical Engineering, The University of North Carolina at Chapel Hill, Chapel Hill, North Carolina, United States of America; 4 Department of Mathematics, University of Florida, Gainesville, Florida, United States of America; 5 Department of Medicine, Division of Pulmonary and Critical Care, University of North Carolina at Chapel Hill, Chapel Hill, North Carolina, United States of America; 6 Center for Environmental Medicine Asthma and Lung Biology, Department of Pediatrics, University of North Carolina at Chapel Hill, Chapel Hill, North Carolina, United States of America; University of Manchester, United Kingdom

## Abstract

In human airways diseases, including cystic fibrosis (CF) and chronic obstructive pulmonary disease (COPD), host defense is compromised and airways inflammation and infection often result. Mucus clearance and trapping of inhaled pathogens constitute key elements of host defense. Clearance rates are governed by mucus viscous and elastic moduli at physiological driving frequencies, whereas transport of trapped pathogens in mucus layers is governed by diffusivity. There is a clear need for simple and effective clinical biomarkers of airways disease that correlate with these properties. We tested the hypothesis that *mucus solids concentration*, indexed as weight percent solids (*wt%*), is such a biomarker. Passive microbead rheology was employed to determine both diffusive and viscoelastic properties of mucus harvested from human bronchial epithelial (HBE) cultures. Guided by sputum from healthy (1.5–2.5 *wt%*) and diseased (COPD, CF; 5 *wt%*) subjects, mucus samples were generated *in vitro* to mimic *in vivo* physiology, including intermediate range *wt%* to represent disease progression. Analyses of microbead datasets showed mucus diffusive properties and viscoelastic moduli scale robustly with *wt%*. Importantly, prominent changes in both biophysical properties arose at ∼4 *wt%*, consistent with a gel transition (from a more viscous-dominated solution to a more elastic-dominated gel). These findings have significant implications for: (1) penetration of cilia into the mucus layer and effectiveness of mucus transport; and (2) diffusion vs. immobilization of micro-scale particles relevant to mucus barrier properties. These data provide compelling evidence for mucus solids concentration as a baseline clinical biomarker of mucus barrier and clearance functions.

## Introduction

**Figure 1:**
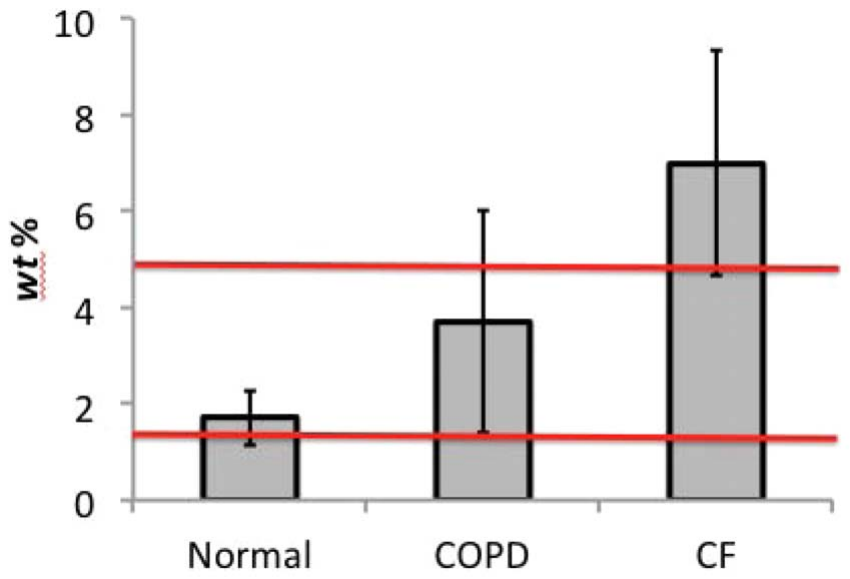
Concentration (*wt%* solids including salts) of sputum for normal, COPD, and cystic fibrosis samples. The data yields: for normal sputum, 1.7±0.56 *wt%* from 17 samples; for COPD sputum, 3.7±2.3 *wt%* from 47 samples; and for cystic fibrosis, 7.0%±2.3 *wt%* from 21 samples. The red lines on the figure at 1.5% and 5% show the range of HBE mucus solids concentrations assayed in this study.

There has been a longstanding observation that *mucus solids concentration* (% solids by weight including salts, denoted *wt%*) rises with increasing severity of many lung diseases such as chronic obstructive pulmonary disease (chronic bronchitis phenotype) and cystic fibrosis (See Figure 1) [1]–[3]. The underlying causes of increased mucus solids concentration are diverse, ranging from depletion of the airway surface liquid due to genetic defects in ion channels as in cystic fibrosis [4] to hypersecretion of mucins in chronic obstructive pulmonary disorders [5], or a combination of both. The consequences of mucus hyper-concentration for disease pathogenesis appear to be reduced mucus clearance and a higher incidence of lung infection [6], [7]. Despite these correlations, an understanding of the explicit biophysical and cell biologic roles for increased mucus solids concentration in airways disease pathogenesis has not been established.

The mucus layer provides host defense for airways by serving as both a barrier to penetration of inhaled materials to the airway epithelium and a vehicle for mechanical clearance. A simple fluid cannot perform these host defense functions. Rather, the requisite flow transport and diffusive properties of airway mucus are attributed to the underlying polymeric backbone generated by the high molecular weight secreted mucins, MUC5AC and MUC5B. Mucins are negatively charged, glycosylated proteins that are continuously synthesized and secreted to replenish the mucus layer. Mucins also contain cysteine-rich domains, where no glycosylation is present, which have hydrophobic properties [8]–[10]. To avoid contact with water, the hydrophobic portions of the molecules form dynamic, physical mucin-mucin interactions that behave as effective crosslinks [11]. Further mucin-mucin interactions result from di-sulfide bonds [12]. The net result of the interactions of mucins with other mucins, as well as other biomolecules present in the mucus layer, is a viscoelastic material that is responsive to a wide frequency range of forcing (breathing, cilia, cough) and to trapped particles whose diffusive paths are controlled by the thermal fluctuations of the mucus molecular network.

The viscoelastic properties of mucus are governed by two frequency-dependent functions: the viscous and the elastic moduli. The relative magnitude of these biophysical functions is highly dependent on the concentration of mucins, and on the distribution of distinct mucin macromolecules. Airway mucus and intestinal mucus, for example, have very different mucin macromolecular distributions. Furthermore, measurable concentrations of DNA and actin macromolecules in airway mucus and sputum are well known in cystic fibrosis and other airways disease, which can confound the role of mucins in biophysical properties [13], [14]. Consequently, the efficiency of mucus clearance (the volume flow rate for a given forcing mechanism) depends on the role and dependence of mucus solids concentration in the interplay between viscous and elastic moduli at frequencies relevant to mucus clearance mechanisms. The three main force clearance mechanisms are: 1) cilia beat-dependent clearance (10–15 Hz); 2) the gas-liquid pumping clearance mediated by tidal breathing (∼0.1 Hz) [15] and 3) cough (a broad frequency spectrum of turbulent air drag). Thus, an assessment of a subject's mucus clearance efficiency and the change in efficiency with disease requires the characterization of mucus viscoelastic properties across a broad range of forcing frequency. Likewise, the diffusivity of mucus, relevant for its barrier properties, is far more complex than that of a viscous fluid, requiring the same level of frequency-dependent information as flow transport (details are given below).

We tested the hypothesis that a simple measure of mucus, concentration (*wt%*) of solids, would serve as a surrogate for the complex biophysics and potentially correlate with both functions of airway mucus: *diffusivity and viscoelasticity*. First, we measured mucus solids concentration (*wt%*) from sputum collected from subjects in our pulmonary clinic and our clinical trial databases; these data establish reference ranges for mucus concentration in health and disease. Second, we used mucus derived from human bronchial epithelial (HBE) cell cultures as a source that could be tuned to *mimic concentrations identified in-vivo* from subject populations. Third, we implemented microrheological particle-tracking techniques [16], [17] in microliter volumes for each *wt%* mucus, using 1 µm particles to approximate the scale of bacteria and drug-delivery particles. This technique both circumvents the volume constraints of airway mucus samples and yields information over a range of physiological frequencies. Fourth, analytical techniques of the datasets provided a comprehensive assessment of barrier (diffusivity) and transport (viscoelasticity) functions of HBE mucus versus concentration (*wt%*). We then investigated the dependence of both diffusive and viscoelastic properties on *wt% of solids*, while also searching for potential signatures of a qualitative transition in either property that could signal a trigger point for disease progression. Our results indicate that both clearance and barrier functions of mucus scale with mucus solids *wt%*, providing a theoretical and practical basis for the utility of mucus solids concentration as a clinically effective marker for phenotyping subjects with airway disease and for outcomes of clinical trials.

## Methods

### Selection of Mucus Model System

The utility of HBE cell culture mucus has been previously demonstrated as both a biochemical and biophysical foundational baseline of normal and pathological pulmonary mucus [18]–[22]. Various biochemical techniques are available, including high-speed density centrifugation, mass spectrometry, combination high pressure liquid chromatography with multi-angle light scattering and refractometry, immunohistochemistry and immunobloting, as well as mass spectrometry. These techniques afford either direct measurements or strong inferences of the biophysical properties of airway mucins such as molecular weight, radius of gyration, and transitions versus concentration into mucin entanglement and reptation regimes [23]–[26]. Further, it was been previously demonstrated that by eluting dilute mucus through columns of differing porosity, in the above mentioned HPLC/light scattering refractometry apparatus, that the % solids make up of HBE cell culture mucus is roughly 1% salt, with the remainder of the % solids divided nearly evenly between mucins and proteins [19].

### Sputum Collection

Sputum was collected from subjects either by spontaneous expectoration or via sputum induction for different protocols. All studies were approved by the UNC Institutional Review Board and informed consent obtained from all subjects. Normal subjects were induced with hypertonic saline for sputum collection; CF and COPD subjects produced both spontaneous sputa as well as receiving induction. The CF and COPD sputa spontaneously expectorated were collected and stored in sterile cups on ice until delivered to the core laboratory. Induced sputum was collected from normal volunteers, CF, and COPD subjects via induction as previously described [20], [27]. In brief, the subjects were given nebulized albuterol followed by nebulized hypertonic saline at increasing doses of 3%, 4%, and 5% until able to produce a sample. All subjects performed throat clearance and nasal clearance prior to producing a sample. If an adequate sample was obtained at the lower doses of hypertonic saline, they did not progress to a higher solids concentration. The sputum samples were kept on ice until delivered to the core laboratory, usually within 30 minutes. Samples were collected in accordance with protocols # 02-1305, 05-2876, and 07-1178, approved by the Office of Human Research Ethics at The University of North Carolina at Chapel Hill. Written consent was obtained from all study participants.

### Weight % Solids Measurements

The solids concentration of mucus was measured by aliquoting between 100 and 200 µL of mucus or sputum on a pre-weighted piece of foil and recording the final mass of the sample and foil. The sample was then placed in an 80°C oven overnight. The final mass of the dried foil and sample was recorded and solids *wt%* calculated [18]. For *in vivo* sputum samples, percent solids (*wt%*) were assessed by measurement of pre- and post-desiccation weights of selected sputum plugs (200–500 mg) [28].

### Preparation of Mucus Samples and Biochemical Characterization

Mucus was harvested from primary human bronchial epithelial (HBE) cell cultures as previously described [18], [19], [21]. Briefly, excess surgical tissue was procured by the UNC Tissue Core Facility. Normal human bronchial epithelial cells were cultured on a 0.4 mm pore-sized Millicell (Millipore, Billerica, MA) coated with collagen and maintained in air-liquid interface media (UNC Tissue Core) as described in [29]. Over a period of 6 weeks, confluent cultures developed cilia, generated and established a periciliary liquid (PCL) layer surrounding the cilia, a mucus layer, and the HBE culture transported mucus. Washings from >100 cultures were pooled and then concentrated against Spectra/Gel to the desired *wt%*. Concentrated mucus was dialyzed against PBS to insure isotonicity as previously described [18], [21].

### Diffusive Microbead Measurements

We selected 1 µm polystyrene particles with carboxyl surface chemistry for use in our assays. This particle size is substantially larger than the length scales of the mucin mesh network [18], [30], [31]. The carboxyl functionalization rather than an amine surface chemistry was chosen as previous studies have shown that amine treated beads have impaired diffusion in sputum [32]. PEG surface chemistries, which enhance the diffusion of smaller particles (200 nm and smaller) [31], [33] in mucus have little effect on the diffusivity of larger (500 nm) particles [33]. These considerations of particle size and surface chemistry imply that the diffusive noise spectrum of 1 µm beads faithfully represents the bulk linear viscoelasticity of the sample.

We note that bead surface treatment as described above is designed to screen the binding affinities that mucins and associated macromolecules in mucus have with certain pathogens and microbes. One must screen these affinities so that the particle fluctuations that are measured are as faithful as possible to the inherent fluctuations of the mucus sample, which are then transformed to viscoelastic moduli by the fluctuation-dissipation relationship of complex fluids. This transformation is described in the Methods sub-section,

### Viscoelastic Transport Characterization

Our sample chamber consisted of a slide and coverslip separated by a double layer of paraffin with a ∼1 cm disc was cut out of the to create a space for 5 µL of mucus to be loaded. Once loaded, mucus samples were imaged by transmitted light, and the motion of diffusing beads was recorded at 60 frames per second with a high- speed video camera (Pulnix; JAI, CA). This frame rate and exposure time were chosen to minimize static and dynamic particle tracking error [34]. Bead position was determined using Video Spot Tracker (Center for Computer Integrated Systems for Microscopy and Manipulation; (http://cismm.cs.unc.edu/downloads/). Between 56 and 178 particles were tracked over 1800 frames at each mucus solids *wt%*.

### Mean Squared Displacement (MSD) and Auto Correlation Function (ACF) statistics of individual particles and ensembles

**Figure 2:**
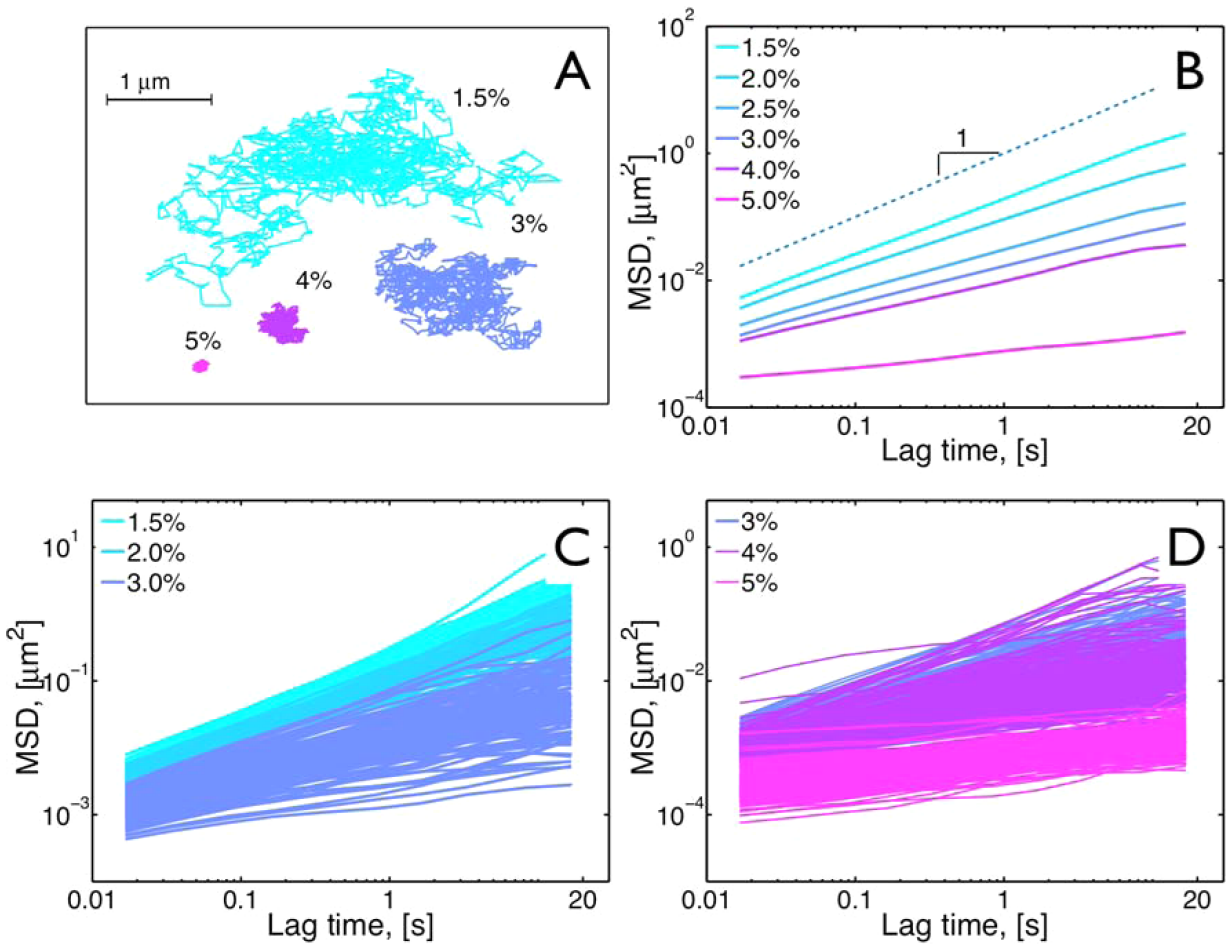
Diffusivity properties of HBE mucus. **A**) Particle trajectories of 1 µm diameter particles for four concentrations over 30 s. **B**) Ensemble-averaged MSD versus lag time for different mucus solids concentrations. The dashed line represents a viscous fluid; any smaller slope indicates sub-diffusive scaling. **C**) Individual or path-wise MSD (iMSD) for particles embedded in mucus samples color-coded by solids concentration, for 1.5, 2.0, 3.0 *wt%*. **D**) iMSD for particles embedded in mucus samples color-coded by solids concentration, for 3.0, 4.0, 5.0 *wt%. Note the vertical scale disparity with* *Figure* **2C**.

Time series of particle positions, 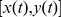 were obtained from the Video Spot Tracker software, typical time series are shown in Figure 2A. Using individual paths (*i.e.*, time positions per particle), the mean squared displacements, 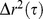 , were calculated as follows,
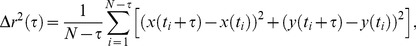
(1)where 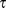 is the time lag and the integer 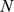 is the total number of frames in a given image stream, which is 1800 for all single particle datasets. The smallest lag is the reciprocal camera frame rate, 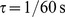, while the largest possible lag is 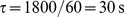. However, statistical significance and independence of data points each limit meaningful plots of MSD to 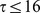 s [34]. The ensemble-averaged MSD, 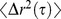 , was calculated as,
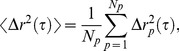
(2)where 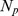 is the total number of particles tracked for a given mucus solids *wt%*.

**Figure 3:**
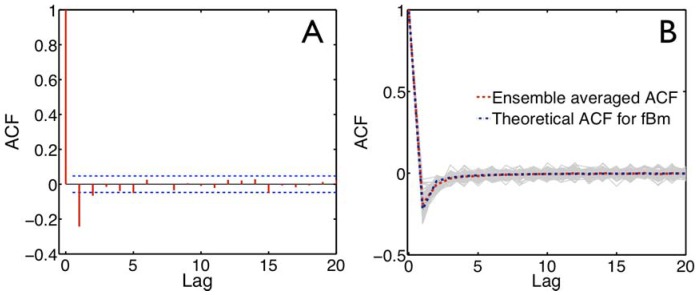
Autocorrelation function data of diffusive particle in mucus. **A**) Typical autocorrelation function (ACF) for a bead diffusing in mucus with 2.5 *wt%* solids. **B**) Individual, ensemble averaged and theoretical ACF for 2.5 *wt%* solids mucus. The equation for the theoretical ACF is given in the Materials and Methods section, from which the value of 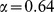 is obtained from Figure 3**A**. This plot is for the ACF in the *x*-coordinate, the ACF in the *y*-coordinate looks similar.

We computed the individual auto correlation function (iACF) by averaging over the *x* and *y* coordinate ACFs (Figure 3). For example, the *x*ACF (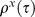) corresponds to correlations in step sizes in the 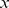-coordinate of a given particle path, 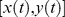, and was calculated as,
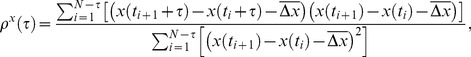
(3)where 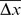 is the average of all the *x*-increments. With the iACF given by 
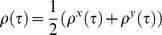
, the ensemble averaged ACF is then 
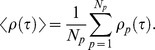
 We note that since time increments were uniform, our ACF is easily scaled to make contact with the velocity autocorrelation function that is routinely reported, cf. [35].

### Diffusive Transport Characterization: Fitting of MSD paths to fractional Brownian motion (fBm)

**Figure 4:**
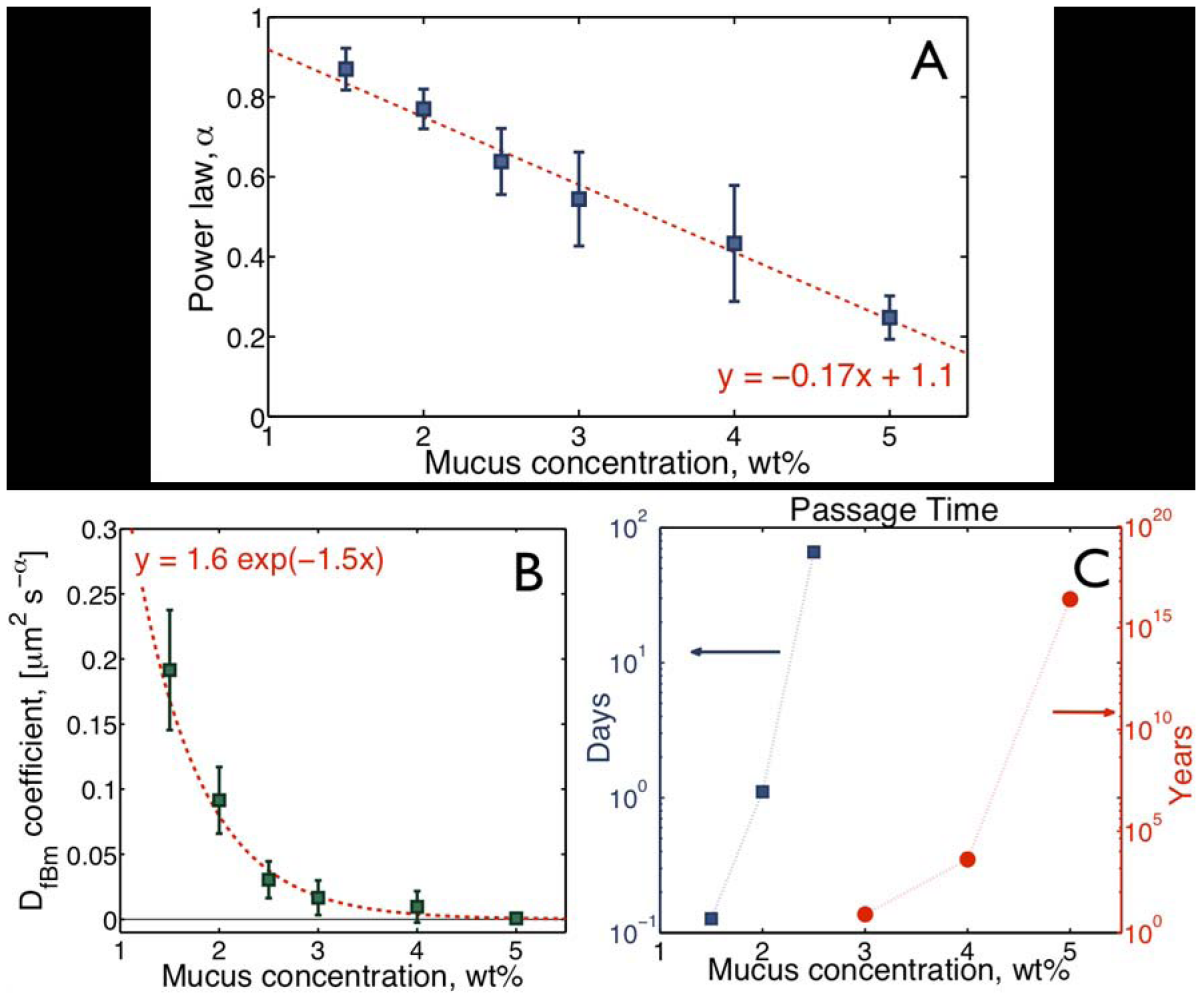
Scaling of the MSD versus mucus solids concentration, where 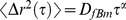. **A**) Power law exponent. Squares represent the averaged values of 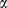 and the vertical bands represent its range over all particle paths. The goodness of fit metric for the linear relationship is 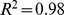. **B**) Scaling of the MSD pre-factor, 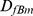, with goodness of fit 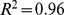. **C**) Rough estimates of mean passage times of 1 micron particles through a 25 micron mucus layer versus *wt%* solids, based on scaling behavior from Figures 4**A&B**.

For all mucus solids concentrations and over the time scales (30 s) of each dataset, we found that the single particle and ensemble MSD data were remarkably well approximated by a uniform power law, as shown in Figure 2 and, therefore, consistent with a scaling of the form,
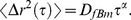
(4)The notation used for the pre-factor, 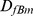, in particular the subscript 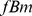, stands for *fractional Brownian motion*, cf. [35]. For all particle path data in mucus, we found 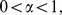 i.e., the particle obeyed uniform sub-diffusive scaling with a consistent exponent 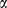 over the entire experimental timescale. Values for 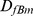 and 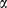 were obtained through standard statistical fitting to linear functions in log-log plots, 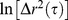 vs 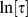 , for 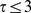 s. These fittings were for single particle displacement data, as opposed to the ensemble average (Figure 4). The reported values of 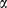 and 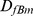 per mucus solids concentration consisted of the mean and spread over all particle paths versus solids *wt%*.

### Viscoelastic Transport Characterization: Transforming MSD statistics to viscoelastic moduli over a broad frequency range

**Figure 5:**
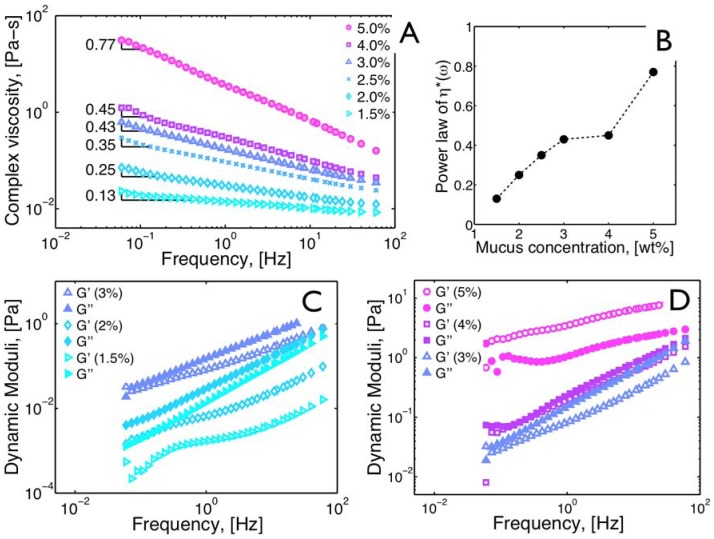
Frequency Dependent viscoelastic properties of mucus. **A**) Frequency-dependent complex viscosity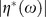 versus frequency 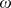 for different mucus solids concentrations. **B**) The slope of the power law, 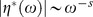, is indicated for each *wt%* solids, both numerically and with a rise vs. run plot. **C**) Storage, 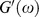, and Loss, 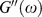, moduli vs. frequency for mucus with 1.5 to 3.0 *wt%* solids. **D**), 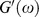
and 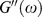 vs. frequency for mucus with 3.0 to 5.0 *wt%* solids.

We follow Mason's protocol [16] to approximate the viscoelastic complex modulus, 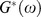 , versus frequency, 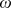 (Figure 5). Using ensemble MSD values, 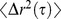 , the complex modulus is given by,
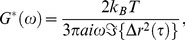
(5)where 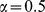 µm is the particle radius and 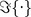 denotes the Fourier transform. The Fourier transform is approximated by:
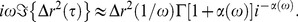
(6)Here 
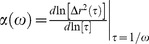
is the local logarithmic slope of 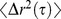 at the frequency 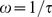 and 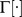 is the Gamma function. This approximation is optimal when the MSD is locally (in lag-time) well-approximated by a power law, which in our data is satisfied not only locally but globally.

### Determining mucus gel-point

**Figure 6:**
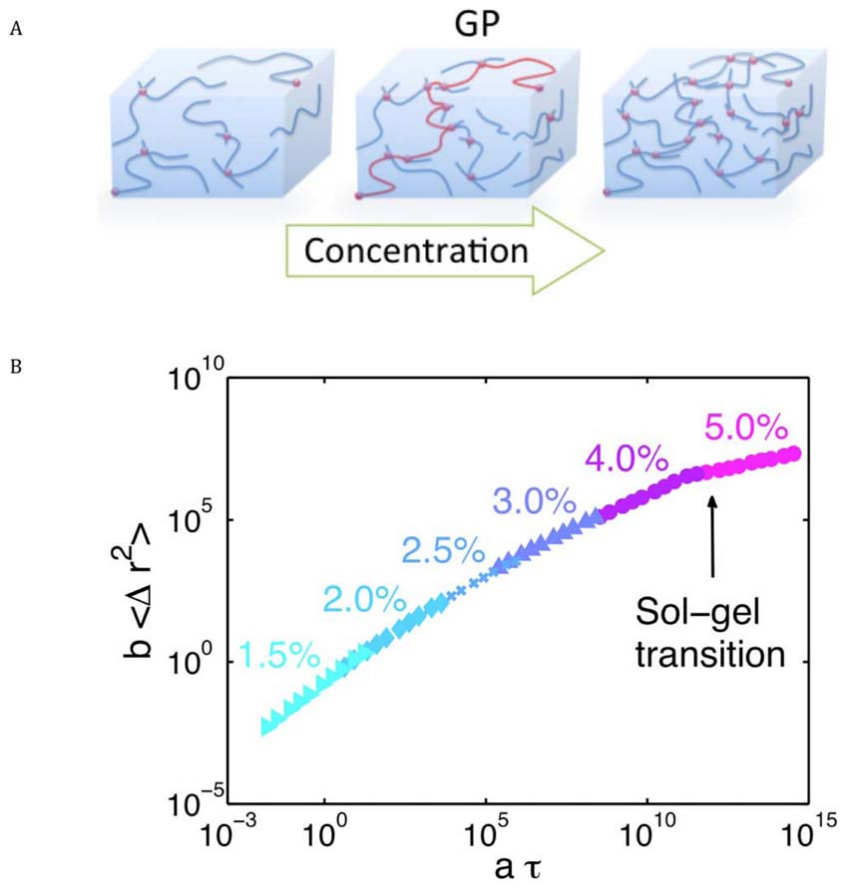
Mucus Gel Point. A) Cartoon illustrating the mucus network changes for increasing macromolecule (mucin) concentration. The gel point (GP) is the point at which the strength of the chains interacting with one another engenders the elastic moduli (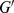) to be comparable in magnitude to the viscous moduli (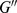). B) Master curve of ensemble-averaged MSDs. The solids concentration for sol-gel transition is obtained following [36], in this case breaking of the slope in the master curve indicates the sol-gel transition occurs at a solids concentration between 4.0 and 5.0 *wt%*.

Potential signatures of qualitative transitions in viscoelastic and diffusive biophysical properties from these microbead rheology tools were also sought. The protocol proposed by Larsen and Furst [36] was employed to detect a signature of a sol-gel transition from the comprehensive MSD data as a function of mucus *wt%*. The point at which this transition occurs is known as the gel point. In the context of 1 µm particles, the gel point was defined as the *wt%* 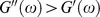 at which mucus underwent a change from a viscous-dominated sol (fluid), for which for all 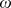, to an elastic-dominated gel, for which for all frequencies 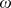. The Larsen-Furst protocol requires scaling each *wt%* MSD curve onto a master MSD curve by scaling the axes by factors *a* (horizontal) and *b* (vertical). The scaled MSD figures versus *wt%*, therefore, have axes 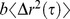 and 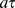, as in [36] and Figure 6. The gel point (GP) is defined as the solids concentration at which the logarithmic slope, or power law, of the shifted master curve “breaks” from one slope below GP to another above GP [36]. Note that these metrics are based on the ensemble MSD scaling behavior versus lag time across multiple samples, rather than the macrorheology standard based on a comparison of 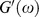 and 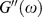 across a frequency spectrum, which require transforms of the MSD data.

## Results

### Mucus Solids Concentration (*wt%*)

Figure 1 shows measured solids concentration (*wt%*) of sputum for normal, COPD, and cystic fibrosis samples. The *wt%* for normal subjects (17 samples from 17 individual patients ranging in age from 20–44, with an average age of 26.6 years) has a mean of 1.7% with a relatively small standard deviation (0.56%). The *wt%* from 28 COPD subjects (47 total samples from patients ranging in age between 52 and 70, average age 60.5 years) were ∼2× higher (3.5%) with a broader range (±2.3%). Sputum *wt%* from 14 CF subjects (21 total samples from patients ranging in age from 24 to 48 years, average 34.8) were ∼4× higher (7.0%) than normal sputum, with a variability similar to COPD (±2.3%).

### Diffusive Transport Characterization

Figure 2 provides a qualitative description of diffusive properties of HBE mucus as a function of solids *wt%*. Figure 2A shows representative trajectories of 1 µm particles in 1.5, 3.0, 4.0 and 5.0 *wt%* mucus. Figure 2B describes the calculated ensemble averaged mean squared displacements (MSDs) for all the mucus solids concentrations investigated, with a dashed line of slope 1 that corresponds to diffusion in a simple viscous fluid. Figures 2C–D show individual time-averaged MSDs. These data reveal an obvious trend toward smaller step sizes as mucus solids concentration increases.

While qualitatively noteworthy, these analyses fail to provide statistically significant data that allow for a meaningful characterization of mucus diffusive properties versus *wt%* of solids. We, therefore, introduced quantitative metrics based upon fitting of individual particle MSD data (Figure 2 C–D) to a fractional Brownian motion (fBm) sub-diffusive law, which relates the squared displacement, 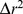 , to the lag-time, 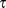, by a two-parameter function given by Eqn. (4).

The use of fBm [37] followed from three basic observations: 1) the increments of the path data were Gaussian; 2) the variance of these increments was stationary over time; and 3) the autocorrelation structure among increments (Figure 3) fitted remarkably well to the theoretical structure of fBm. A similar argument was used in modeling the motion of fluorescently labeled bacterial chromosomal loci in cytoplasm [35], and fBm as a model can be supported by other statistical techniques [38], [39]. Note that for normal diffusion (simple Brownian motion) of a particle of radius 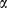 in a fluid of viscosity, 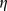, the MSD exponent is 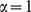 and the pre-factor, 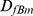 , reduces to the diffusion coefficient 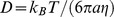 . Therefore, any information pertinent to particle size and fluid viscosity is included in the pre-factor. For sub-diffusion, and fractional Brownian motion in particular, little is known about the physical basis for the pre-factor, and it has therefore been relegated to a secondary feature of fBm in the literature. We found, however, that both fBm parameters correlated remarkably well with mucus *wt%*.

The first striking result of Figure 4A is the linear scaling behavior of the power law exponent versus mucus solids concentration:
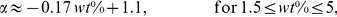
(7)with a very high confidence level, 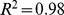. The second feature of note in Figure 4A is associated with the spread in the fitting (error bars), which was relatively low at 1.5 and 2 *wt%*, grew at 3 *wt%*, grew again at 4 *wt%*, but then dropped significantly at 5 *wt%*. This feature, a sharp maximum in the spread among individual particle paths at a fixed *wt%*, is suggestive of a transition in the microstructure of mucus. At 4 *wt%*, there were many more “fast” and “slow” particle outliers relative to the mean. Yet at 5 *wt%*, not only was the behavior more strongly sub-diffusive (a drop in 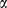) but there were very few outliers, with all particles essentially immobilized. This behavior is consistent with a sol-gel transition. Similar to our results, a spread of MSD data was recently observed in respiratory mucus samples [40] and suggested as an indicator of heterogeneity in pore size in the mucus microstructural network.

As indicated in Figure 4B, we found a robust trend in the pre-factor 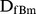 versus mucus solids concentration, in this case with an exponential form:
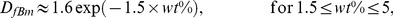
(8)and once again with a high 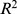 value of 0.96.

The fittings given by Eqns. (7)–(8) have immediate implications for the expected particle passage time, denoted 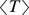 , through a mucus layer of thickness, 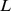. While first passage time properties are not understood for general subdiffusive processes, the self-similarity property of fBm allows us to compute reasonable order-of-magnitude estimates for our physical processes. Let 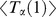 denote the mean passage time of fBm to exit from the interval 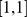 starting from the origin. It follows that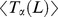, the mean exit time from the interval 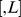, is 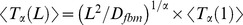 [41]. There are, as yet, no analytical results for determining 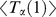, but numerical evidence reveals that this is order 1 for the values of 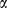 we are interested in here. In this manner, an estimate for the mean passage times of 1 micron diameter particles through a 25 micron layer was calculated and graphed in Figure 4C. The graph reveals a dramatic disparity in mean passage times through a nominal airway mucus layer between 1.5 *wt%* and 5 *wt%* mucus, i.e., from minutes at 1.5 *wt%* to complete immobilization at 5 *wt%*. We caution that these estimates are crude indicators of passage times versus mucus solids concentration prior to full-scale direct numerical simulations, but the essential conclusion is clear and compelling.

### Viscoelastic Properties

The viscoelastic properties of HBE mucus were calculated from the MSD statistics, following the protocol proposed by Mason [16]. Figure 5A shows the amplitude of the frequency-dependent complex viscosity 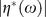 for 1.5, 2.0, 2.5, 3.0, 4.0, 5.0 *wt%* mucus. The uniform power law behavior of MSD versus lag time, illustrated in Figure 2B, was recapitulated as a uniform power law in the complex viscosity in frequency space, 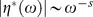, where the exponent 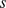 decays with decreasing solids concentration (Figure 5A). However, in this case there was not a clear functional form of the frequency-space exponent 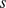 versus *wt%*, as shown in Figure 5B.

The real and imaginary parts of the complex viscosity carry detailed information about the loss (viscous) modulus, 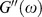, and storage (elastic) modulus, 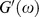, at each frequency, where 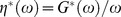 and 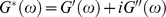. Thus, since Figure 5A did not inform the relative contributions of the storage and loss moduli in the scaling behavior, 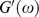 and 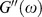 were plotted separately for each *wt%* in Figures 5C–D. The graphs are color-coded and font-specified by *wt%*, with 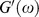 denoted by empty symbols and 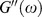 denoted by filled symbols. Several striking results are conveyed by Figures 5C–D.

For example, below 3.0 *wt%*, the loss modulus, 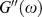, dominated the storage modulus, 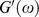, uniformly across all frequencies, implying low *wt%* mucus is a viscoelastic solution, or sol, throughout this frequency range (Figure 5C). At 3.0 *wt%*, the curves of 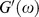 and 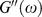 approach each other over the full frequency range.

At 4.0 *wt%*, the elastic and viscous moduli were almost equal over a frequency range between 0.1 and 10 Hz, suggesting the *onset of a transition from solution-like to gel-like behavior*. Over the remaining frequency range, the gap between the larger viscous and lower storage modulus was significantly less than the lower *wt%* data, again suggestive of the onset of a transition. At 5.0 *wt%*, the elastic modulus, 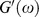, dominated the viscous modulus, 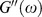, over the full frequency range implying *high solids wt% mucus is a viscoelastic gel* at frequencies above 0.1 Hz.

As summarized in the Methods section, quantitative metrics have been developed to characterize the sol-gel transition [36], [42] from passive microbead data. These MSD-based metrics were implemented in Figure 6B, which revealed evidence for a mucus sol-gel transition, or the point at which the elastic modulus (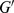) is larger in magnitude that the loss modulus (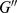), just above 4.0 *wt%*.

**Figure 7:**
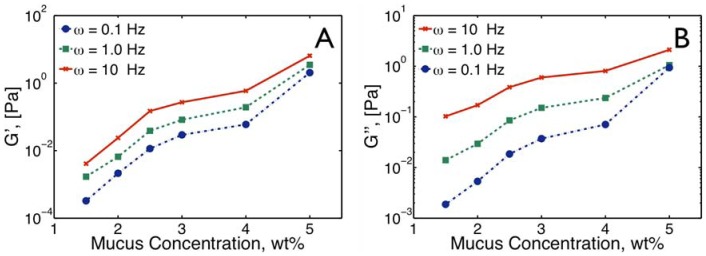
Concentration dependent viscoelastic properties of mucus at key frequencies. **A**) Elastic (storage) modulus, 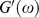, versus mucus solids concentration for three representative frequencies (from cilia to tidal breathing). **B**) Viscous (loss) modulus, 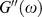, versus mucus solids concentration for three representative frequencies.

Next, we related our experiments to reported viscoelastic studies of mucus by extracting the dynamic moduli at the following frequencies: 0.1 Hz to approximate the frequency of tidal breathing; 10 Hz to approximate the frequency of the cilia beat cycle; and, 1 Hz as an intermediate frequency added simply to bridge the 0.1 and 10 Hz data. Figure 7 shows the storage modulus 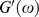 (Figure 7A) and loss modulus 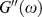 (Figure 7B) versus *wt%* for 0.1 Hz (black dots), 1 Hz (green squares), and 10 Hz (red x). The salient features of Figure 7 can be summarized in the following points.

First, the capacity of mucus to store energy at a given frequency of forcing is determined by the elastic (or storage) modulus,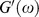. Vertical slices of data plotted in Figure 7A revealed that 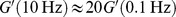 for 1.5–4.0 *wt%*, i.e., the ratios of the elastic moduli relevant to cilia and tidal breathing remain constant over this range of *wt%*, even though the moduli are growing significantly. Thus, across these solid concentrations, mucus is tuned to preferentially store energy from cilia forcing (10 Hz) more so than from tidal air drag (0.1 Hz). This observation implies that mucociliary clearance exploits the elasticity of the mucus layer far more so than air drag. Conversely, at disease-associated mucus solids concentrations of ∼5 *wt%*, the differentiation in energy storage vs. frequency is diminished (the curves in Figure 7A are converging).

Second, comparison of the change in storage modulus 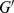 from the lowest (1.5 *wt%*) to the highest (5.0 *wt%*) mucus solids concentration revealed that the storage modulus associated with cilia forcing (10 Hz) increased by a factor of 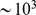 , whereas the storage modulus for tidal breathing (0.1 Hz) increased by a factor of 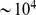 . This dramatic increase in elastic modulus with *wt%* suggests that at high *wt%* mucus is resistant to shear deformation from all physiological phasic stresses, including single cilia, ciliary carpets, and air drag. Importantly, this striking increase of 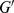 in the 10–15 Hz range implies individual cilia would be unable to generate sufficient force to penetrate the mucus layer.

Third, the energy dissipated by mucus at forcing frequency 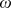 is measured by the viscous (or loss) modulus, 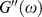. Vertical slices of the data shown in Figure 7B revealed *wt%* dependent degrees of differentiation at the 10 Hz of cilia relative to the 0.1 Hz of tidal breathing. The differentiation was most significant in low *wt%* mucus and decreased with increased *wt%*: 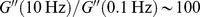 at 1.5 *wt%*, ∼70 at 3.0 *wt%*, ∼30 at 4.0 *wt%*, and ∼5 at 5.0 *wt%*. These data imply that healthy *wt%* mucus dissipates relatively less energy from tidal breathing than from ciliary beating. This observation reinforces the implication from the storage modulus results that cilia-dependent clearance is tuned more to elasticity whereas clearance mediated tidal breathing, i.e. “gas-liquid pumping”, is tuned more to the viscous component of healthy mucus. These differentiated moduli then disappear at high solids *wt%* typical of advanced airways disease.

Fourth, the absolute changes in viscous moduli versus *wt%* were strongly frequency dependent. 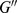 at cilia beat frequency rose by a factor of 10–20 across the 1.5 to 5 *wt%* range, whereas at tidal breathing frequency, 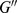 rose by a factor of ∼600. These data imply cilia-induced viscous stress dissipates 10–20 times more rapidly in unhealthy vs. healthy *wt%* mucus, whereas tidal breathing stress dissipates 100's of times more rapidly in unhealthy mucus. The implication is that the previously noted viscosity-dominated emphasis of the tidal breathing (“gas liquid pumping”) clearance mechanism at healthy *wt%* mucus is precipitously lost with increased *wt%*.

## Discussion

Abnormal mucus solids concentration, measured as *wt%* (solids), has been associated with airways disease, but has not been developed as a biomarker for lung disease because its pathophysiologic relevance has remained unclear. Recent evidence has suggested that the high molecular weight airway mucins in mucus form an interpenetrating mesh whose biophysical functions are highly dependent on their solids concentration, i.e., their hydration [22]. Using microrheological experimental approaches and new analytic techniques, we have systematically characterized the diffusive and viscoelastic properties of HBE mucus that relate to its barrier function and transportability over normal (1.5–2 *wt%*) and disease associated (>4 *wt%*) solids concentrations and physiologically relevant frequency spectra.

With respect to the barrier properties of mucus, the fate of inhaled particles was modeled from measurements of the diffusive properties of particles in airway mucus. A particle size was selected that was relevant to disease, i.e., 1 µm particles, mimicking bacterial size. It is well known that the motion of microscopic particles in complex liquids and soft gels is described by a mean-squared-displacement that is sub-linear in time (called sub-diffusive) [16], [25], [43]–[45]. However, predictive simulations of the passage times of particles through mucus at varying solids concentrations are only possible once accurate sub-diffusive laws and best-fit parameters are determined, since there is no theoretical basis (i.e., no analytical formula) for passage time distributions of sub-diffusive stochastic processes. In this work, we fitted fractional Brownian motion [35], [37]–[39] to comprehensive experimental data and demonstrated a remarkably robust fit and scaling of the model parameters versus mucus solids *wt%* (Figure 4). Consequently, we have identified an accurate sub-diffusive law and found best-fit parameters for the ensemble particle path data in each *wt%* mucus.

Figures 2 and 4 provide a robust characterization of the diffusive properties of HBE mucus versus solids concentration that demonstrate four critical points. First, at every fixed mucus solids *wt%*, individual path and ensemble averaged MSD versus lag time were linear on a log-log scale, indicating a uniform power law behavior that was remarkably consistent with fractional Brownian motion, Eqn. (4). Second, beyond exhibiting a power law scaling in the MSD, path increments were Gaussian and the autocorrelation function (ACF) exhibited remarkable agreement with the theoretical form for fractional Brownian motion, (Eqn. (3) and Figure 3). Third, the two data-inferred parameters that characterize fractional Brownian motion, the power law exponent 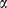 and the pre-factor 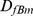 , obeyed robust fits to a linear and an exponential dependence on mucus *wt%*, Eqns. (7)–(8), respectively. Fourth, both fBm parameters, 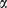 and 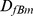 , were decreasing functions of mucus solids *wt%*, indicating sub-diffusivity was progressively exaggerated as mucus solids concentration increased.

This scaling behavior of MSD translated to a rough estimate of mean passage times for a 1 micron particle, the approximate size of airway bacteria, through a 25 micron mucus layer as: hours at 1.5 *wt%*; 1 day at 2.0 *wt%*; months at 2.5 *wt%*; a year at 3.0 *wt%*; and, effectively complete immobilization at 4.0 *wt%* and above. There was a signature just above 4.0 *wt%* indicating both a smaller sub-diffusive exponent and a dramatic reduction in variability across paths. Above 4.0 *wt%*, there were essentially no outliers, implying complete immobilization of trapped micro-particles.

The data described in Figure 5 point to an explanation for this observation. A clear transition from 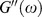 -dominated moduli (a viscous-dominated solution) to 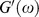 -dominated moduli (an elasticity-dominated gel) was observed at a critical mucus *wt%* over the full frequency range. This behavior is the classical macro-rheological signature of a sol-gel transition [42]. The *gel point* was more accurately inferred by comparing the MSD statistics of passive microbead data obtained from samples that were in both the sol (or solution) and gel phases (Figure 6). These conditions are easily observed in real time during observations of a gelation process as discussed in [36].

We note the similarities between our findings on HBE cell culture mucus versus *wt%* of solids with recent findings of Georgiades *et al.* on purified gastrointestinal mucins versus mucin concentration [23], [26]. Namely, Georgiades *et al.* identify a concentration-dependent, power law scaling in the viscosity of purified mucin below a threshold concentration, followed by a transition to a different power law scaling above a concentration threshold. Thus, a key macroscopic metric (viscosity) of purified gastrointestinal mucins strongly correlates with mucin concentration, marked by a qualitative transition at a critical concentration that is associated with an entanglement transition and an important role of reptation. We note the related work of Katz et al. [46] where viscosity of CVM was shown to scale with hydration, the work of Verdugo et al. [47] where the role of ion-dependent swelling of mucus gels affects viscoelasticity. Our studies are on HBE mucus, which not only contains mucins but additional proteins that constitute the mucus network; our qualitative transition at a critical *wt%* of solids is a gel transition where a spectrum of viscous and elastic moduli switch relative dominance. As a further point of comparison of these studies, the low frequency viscosity of HBE cell culture mucus at any fixed *wt%* solids is significantly higher than the viscosity of purified mucins at a comparable mucin concentration. This underscores the critical role of the additional proteins in mucus beyond pure mucins in constituting the mucus network and biophysical properties. Furthermore, this purified mucin vs. cell culture mucus comparison points to additional studies necessary on cell culture mucus vs. clinical mucus and sputum samples, to identify the biophysical and viscoelastic impact of additional proteins, as well as other biomolecules including actin and DNA [13], [14].

Once we determined the gel point (GP) of HBE mucus to be between 4 and 5 *wt%* (Figure 6), the physical basis for the observations in the MSD shown in Figures 2 and 3 became apparent (see descriptive model, Figure 6A). As described above, mucus contains large hydrogel-forming mucins, which together with other proteins form a mesh that provides mucus its viscoelastic properties. At low *wt%* solids, the spatial structure of the mucin network is relatively homogeneous at micron scales, i.e., all particles exhibit similar sub-diffusive behavior as indicated by the relatively small error bars seen in Figure 4. Just below the *wt%* of the GP, the network forms micro-domains that increase in density as the GP is approached, i.e., the particle ensemble experiences diverse microenvironments resulting in a larger spread of the MSD power law. Above the GP, the gel domains dominate the mucus network so that all particles experience highly confined behavior with a reduction in the MSD power law, 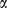, and pre-factor, 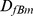, and a reduction in the spread across the particles. Our analysis should facilitate future studies to explore the interactions of mucus solids concentration with a spectrum of particle sizes and surface chemistry.

The measured decrease in micro-particle diffusivity, as a function of increasing mucus *wt%* solids, may reflect an enhanced mucus barrier function with disease. Indeed, this response could be beneficial compensation to buffer the exposure to environmental stressors associated with environment-induced airways disease. However, it has been reported that bacteria deposited on high *wt%* mucus favor biofilm formation, in part because of decreased mobility of bacteria and diffusivity of auto-inducers from the point of bacterial deposition [18].

Our data also provided insights into the relationships between *wt%* solids, viscoelastic properties, and mucus transport. The physical mechanisms and magnitudes of force application to produce transport of mucus are quite different for each transport mechanism. For example, force application by cilia requires rapid (10 Hz) penetration of cilia into the mucus layer mesh and mesh deformation. Gas liquid pumping requires that the force of airflow be imparted to mucus by an interaction of air and the mesh dependent “roughness” of the air-exposed face of the mucus layer. Cough produces a more turbulent, high frequency form of air drag on the mucus layer.

The present work (Figures 5–7), and previous studies in cystic fibrosis sputum (both whole [24], and samples separated by low speed centrifugation [48]), cervicovaginal mucus (CVM) [49], sino-nasal mucus in patients with chronic sinusitis [50], and pig gastrointestinal mucus (PGM) [51], [52], demonstrate that single frequency viscoelastic moduli are insufficient to draw inferences about the efficacy of mucus transport mechanisms. The data reveal that the viscous and elastic moduli of a given mucus sample vary by orders of magnitude across the frequency spectra relevant to each mode of mucus transport. Importantly, an even greater variation is observed versus mucus solids *wt%*. Such widely varying mucus solids concentrations require tools in place that detect the physiologically relevant viscoelastic properties of a mucus sample specific to each clearance mechanism.

A complete biophysical understanding of the contribution of viscoelastic properties to the variation in mucus transport in patients is not available. However, we note that a correlation between concentrated, pathological mucus and decreased clearance has been reported [1] and our data yields insights into the mechanisms by which solids *wt%*-dependent changes in viscoelastic properties may contribute to this correlation. For example, Puchelle [53] demonstrated that when cilia beat against a viscosity higher that 100 mPa·s, i.e., a viscosity 100 times that of water, beat frequency decreased and mucus clearance slowed. More recently, direct force measurements of individual airway cilia revealed that cilia are tuned to beat against a 100 mPa·s fluid, but begin to fail at higher viscosities [1]. Our results show that mucus in excess of 2.5 *wt%* solids exhibits complex viscosities higher than the 100 mPa·s threshold at frequencies characteristic of cilia (10 Hz), providing key data linking increased mucus concentrations to the observation of Puchelle *et al.* [53]. Our results further predict reductions in mucus clearance efficiency with increased mucus solids *wt%* across the entire physiological frequency force spectrum, from the 0.1 Hz of tidal breathing to the 10 Hz of cilia beat, determined on the basis of viscous and elastic modulus transitions (Figure 5). Combining our results with previous studies, we conclude that cilia dependent and gas liquid dependent mucus clearance is significantly and progressively compromised at mucus concentrations above 2.5 *wt%* solids.

Cough is the principal back-up mechanism for failed mucus clearance, and our viscoelastic analyses also provide insight into the effectiveness of cough to offset concentration-dependent inefficiencies in cilia and breathing-induced mucus transport. For example, cilia have to penetrate the mucus layer for their force to be transferred to the layer and induce transport. Under high *wt%* solids conditions where cilia cannot transfer force to mucus, the 20-fold increases in 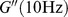 and 100-fold increases in 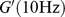 between healthy and unhealthy solids *wt%* mucus could be temporarily reduced by the violent air drag associated with cough. Sufficient forcing from cough has the potential to shear-thin (lower 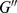) and soften (lower 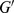) mucus, affording cilia a brief window to recapture their ability to penetrate the mucus layer before the high solids *wt%* mucus gel recovers from the disruptions induced by cough. Some airways diseases, e.g., in particular CF, lead to continuous cough, presumably in part to generate more efficient transport properties of mucus.

Another mechanism for compromised mucociliary clearance (MCC) associated with pathological, high solids concentration mucus has been recently identified, namely an increase in the relative osmotic pressure of the mucus layer versus the gel-like properties of a concentrated periciliary layer. The osmotic pressures of each layer reflect the activities of the interpenetrating, i.e., semi-dilute, mesh-like properties of their respective mucins. Button *et al.* [22] demonstrated that the mucus and periciliary layers must be in osmotic balance for efficient MCC. When the osmotic pressure of the mucus layer exceeds that of the periciliary layer, water is drawn from and collapses the periciliary layer, compromising the normal cilia beat stroke.

By comparing the mucus layer's osmotic modulus vs. periciliary layer height data with the measured osmotic modulus of mucus vs. *wt%* (data reported by Button *et al.*, Figures 6 and S1 [22]), the osmotic pressure of mucus began to exceed that of the periciliary layer at a mucus concentration of ∼5 *wt% (a value approaching that seen in CF mucus)*, causing a decrease in periciliary layer height. At concentrations at and above 8.0 *wt%*, the periciliary layer was completely collapsed, totally disabling MCC. These findings are complementary to our results, which predict the onset and progression of cilia and tidal breathing-induced mucus transport at much lower mucus solids *wt%*, beginning between 2.5 and 3.0 *wt%*. Further, we found that a dramatic transition in mucus viscoelasticity across all physiological frequencies arose at ∼4.0 *wt%*, associated with a sol-gel transition (Figure 6). Thus, our data predict a slowing of mucus transport due to abnormal viscoelastic properties prior to complete osmotic collapse of the periciliary layer and shutdown of mucociliary clearance. Indeed, slowing of mucus transport has been observed in COPD subjects with mucus solids *wt%* in the 2–4% range [54].

Collectively, our data suggest that the *wt%* solids of mucus will serve a simple surrogate for the mucin concentrations that govern the key viscoelastic properties of mucus relevant to flow/no-flow. One question is whether *wt%* solids will perform well in sputum from subjects with airways disease. In CF sputum, the uniquely large amount of DNA and actin, with its intrinsic viscoelastic properties in extracellular solutions, may confound the *wt%* solids measurement, and likewise confound the source of potentially dramatic changes in viscoelastic properties. Precise measures of the *wt%*-dependent DNA effects on CF sputum biophysical properties will be required to answer this question. Importantly, the presence of inflammatory cells contributes negligibly to *wt%* over a wide range of cell numbers, suggesting *wt%* will be useful in subjects with several inflammatory airways diseases, e.g., both COPD and CF. Indeed, *wt%* solids may be a particularly useful biomarker to identify subsets of subjects with COPD that exhibit a chronic bronchitic phenotype.

In summary, the significant conclusions of this study lie in the robust scaling of both diffusive and viscoelastic properties of HBE cell culture mucus versus *wt% of solids*, ranging from those associated with normal sputum concentration to pathological concentrations. Our analysis using fractional Brownian motion, and in particular Eqns. (7) and (8), offers an efficient protocol to access this information, and it provides compelling evidence that the simple marker of mucus *wt%* solids is an indicator of the diffusive and viscoelastic biophysical abnormalities of mucus associated with disease. These findings point to *wt%* solids of lung mucus as a candidate for a rational and easily applied clinical biomarker of airways disease.
