# Correction: A Biophysical Basis for Mucus Solids Concentration as a Candidate Biomarker for Airways Disease

**DOI:** 10.1371/journal.pone.0097980

**Published:** 2014-05-15

**Authors:** The PLOS ONE Staff

There were errors in the Funding section. The correct funding statement is as follows: This research was funded by National Science Foundation grants DMS-1100281, DMR-1122483, National Institutes of Health grants NIH/NHLBI 1 P01 HL 108808-01A1, NIH/NHLBI 5 R01 HL 077546-05, P01-HL-118073, a Simons Foundation Collaboration Grant Number 245653, and the Cystic Fibrosis Foundation (HILL08I0, BUTTON07XX0). The funders had no role in study design, data collection and analysis, decision to publish, or preparation of the manuscript.
